# Analysis of Sour Porridge Microbiota and Improvement of Cooking Quality *via* Pure Culture Fermentation Using *Lacticaseibacillus paracasei* Strain SZ02

**DOI:** 10.3389/fmicb.2021.712189

**Published:** 2021-08-26

**Authors:** Cheng Wang, Yunhe Xu, Bin Yu, Aibo Xiao, Yuhong Su, Haonan Guo, Huajiang Zhang, Lili Zhang

**Affiliations:** ^1^Department of Food Science and Engineering, Jinzhou Medical University, Jinzhou, China; ^2^Department of Food Science and Engineering, Qilu University of Technology, Shandong Academy of Sciences, Jinan, China; ^3^Liaoning Agricultural Development Service Center, Shenyang, China; ^4^Department of Food Science, Northeast Agricultural University, Harbin, China

**Keywords:** broomcorn millet, fermentation, *Lacticaseibacillus paracasei*, *Panicum miliaceum*, peak viscosity, sour porridge

## Abstract

The microbial composition of sour porridge at different fermentation times was analyzed through high-throughput sequencing, and a pure culture fermentation process was established to optimize production process and improve the edible quality of the porridge. In natural fermentation, Firmicutes and Proteobacteria were abundant throughout the process. Specifically, *Aeromonas, Acinetobacter*, and *Klebsiella* were dominant on fermentation days 1–5 (groups NF-1, NF-3, and NF-5), while *Lactobacillus* and *Acetobacter* gradually became the dominant bacteria on fermentation day 7 (group NF-7). Further, we isolated one strain of acid-producing bacteria from sour porridge, identified as *Lacticaseibacillus paracasei* by 16SrRNA sequencing and annotated as strain SZ02. Pure culture fermentation using this strain significantly increased the relative starch and amylose contents of the porridge, while decreasing the lipid, protein, and ash contents (*P* < 0.05). These findings suggest that sour porridge produced using strain SZ02 has superior edible qualities and this strategy may be exploited for its industrial production.

## Introduction

Fermented grain porridge offers nutritional benefits derived from the grain itself as well as from the probiotic properties brought about by fermentation. Moreover, fermentation also degrades certain anti-nutritional factors contained in grains, such as phytate and tannins. Therefore, fermented grain porridge has become a popular food item worldwide. For example, Ogi in West Africa is a popular local dish and is also the main food provided to newborns after weaning. Similarly, fermented grain porridges such as Ben-saalga in Burkina Faso and Togwa in Tanzania play important roles in local recipes and culture (Melini et al., 2019). Sour porridge is a traditional fermented cereal food popular in the north-western part of inner Mongolia and the north-western part of Shanxi in China. It is prepared by natural fermentation of broomcorn millet (*Panicum miliaceum*) in an acidic liquid. Its popularity among local consumers is attributed to its smooth taste, unique sour flavor, and bright yellow color (Wang Q. et al., 2019). The natural fermentation procedure includes cleaning the broomcorn millet by washing and draining, adding water, and incubating the mixture for at least 24 h. The acidic liquid is decanted, fresh water added, and boiled for 15–20 min (Supplementary Figure 1). A longer cycle culture time of acidic liquid enhances the flavor of sour porridge.

The microorganisms in the acidic liquid play a key role in the fermentation process. The microbial community involved in the natural fermentation process is dynamic, and its composition is determined by the four major ecological processes: diversification, dispersal, selection, and drift (Danczak et al., 2020). The microbial community composition is also greatly affected by human activity, climate, humidity, and geographical location. The microbiota of naturally fermented sour porridge was previously analyzed using culture-independent technologies such as polymerase chain reaction denaturing gradient gel electrophoresis (PCR-DGGE) and high-throughput sequencing, and *Lactobacillus* was identified as the dominant genus (Wang et al., 2018). Further, Chen et al. (2002) isolated and cultured a large number of *Lactobacillus* species from naturally fermented sour porridge using culture-dependent methods. Microorganisms can improve the edible quality of grains by changing their physical and chemical properties. For example, naturally fermented corn starch has a higher gel strength and produces stronger noodles with a smoother appearance than unfermented corn starch (Yuan et al., 2008). Natural fermentation removes few proteins and lipids in the rice flour, thereby changing the rheological properties of the rice flour. The noodles made from fermented rice flour have better chewiness and reduced mineral content, which provides a whiter and brighter appearance (Lu et al., 2003). Sweet potato starch fermented by *Lactiplantibacillus plantarum* has a higher rate of water retention and expansion and increased peak viscosity, yielding highly transparent noodles with an elastic consistency that is not easy to break during cooking in boiling water (Liao and Wu, 2017). Fermentation by *L. plantarum* NRRL B-4496 changes the hydrophobicity of the surface protein in pea flour, altering the emulsification and foaming properties and conferring new processing characteristics which have different uses in food processing (Çabuk et al., 2018). Fermentation of millet causes the destruction of the crystalline structure of starch granules; this destruction decreases the pasting onset temperature and enthalpy, making pasting easier and increasing the resistant starch content (Amadou et al., 2014; Xu et al., 2020). Despite these studies on starch microbial fermentation, there is a gap in the understanding of the microbiota and specific strains involved at different stages in the fermentation of sour porridge, including the optimal time of product maturity. Additionally, the mechanism of *Lactobacillus* fermentation is not clearly known. These gaps have led to the inconsistent quality of sour porridge products and difficulties in guaranteeing product safety, thus restricting the industrial production of sour porridge.

In this study, we analyzed the microbiota of the naturally fermented sour porridge with the aim of characterizing the microbial profile at different stages of fermentation and establishing a pure culture fermentation strategy to optimize production and improve edible quality of the product. We used high-throughput sequencing to determine the bacterial composition of sour porridge at different stages of natural fermentation. Finally, a high content of acid-producing *Lactobacillus* strain was isolated, sequenced, and investigated for its effects on cooking quality and pasting properties of the porridge. This work is intended to provide a technical reference for the large-scale industrial production of high-quality sour porridge.

## Materials and Methods

### Preparation of Sample

Approximately 50 g of broomcorn millet was fermented by soaking in 450 mL water and incubated at room temperature (25–30°C) for 24 h. From this sample, 25 g of fermented broomcorn millet and 100 mL of fermentation broth were removed. Meanwhile, rice soup was prepared by boiling 25 g of raw grains with 250 mL of water for 15 min. From this sample, 25 g of raw grains and 100 mL of rice soup were added into the acidic liquid (fermentation broth) from the previous fermentation for further incubation. This procedure was repeated daily, samples were collected after 1, 3, 5, and 7 days, and were named NF-1, NF-3, NF-5, and NF-7, respectively. Each group was analyzed in triplicate, and samples were preserved in liquid nitrogen prior to sequence analysis.

### Analysis of Microbial Composition and Metabolism

Microbial genomic DNA was extracted from 12 samples using a TIANamp Stool DNA Kit (TIANGEN Biotech, catalog #DP328). The purity and concentration of extracted genomic DNA was examined by agarose gel electrophoresis. The hypervariable V3–V4 region of the 16S rRNA gene was PCR amplified using the forward primer 338F (5′-ACTCCTACGGGAGGCAGCA-3′) and the reverse primer 806R (5′-GGACTACHVGGGTWTCTAAT-3′). Amplified sequences were purified, quantified, and sequenced on an Illumina Novasep PE250 platform by Personal Biotechnology Co., Ltd. (Shanghai, China). The raw data derived from the sequencing process was processed using the DADA2 method to obtain amplicon sequence variants (ASVs) on the QIIME2 platform, and subsequent analysis was based on ASVs. The Chao, Shannon, and Simpson indices were computed using the MOTHUR software. Non-metric multidimensional scaling (NMDS) analysis was performed with the vegan package in R. Functional bacterial genes in sour porridge were predicted using the PICRUSt2 program. The functional genes were mapped to the Kyoto Encyclopedia of Genes and Genomes (KEGG) database, and the relative abundance of genes related to metabolic pathways in groups NF-3 and NF-7 were annotated.

### Isolation and Identification of Bacterial Strains

Bacterial strains were isolated by plate dilution. Serial dilutions of sour porridge samples were spread onto rice soup solid medium plates containing 2% w/v glucose, 2% w/v agar, and 2% w/v soybean oligopeptide (Nanjing Deju Biotechnology Co., Ltd., Nanjing, China) and incubated at 30°C for 48 h under aerobic conditions. The bacterial colonies obtained from the plates were purified using the streak plate method until pure cultures were obtained. After purification, bacterial colonies were inoculated into a liquid medium of rice soup containing 2% w/v glucose and incubated at 30°C for 24 h. Screening for acid-producing strains was performed by measuring the pH value of the rice soup fermentation broth. The bacterial genomic DNA was extracted using a TIANamp Stool DNA Kit. Full-length 16S rDNA of bacteria was PCR amplified using the forward primer 27F (5′-AGAGTTTGATCCTGGCTCAG-3′) and the reverse primer 1492R (5′-CTACGGCTACCTTGTTACGA-3′). Amplified sequences were purified and sequenced using an ABI 3730XL automated sequencer (Personal Biotechnology Co., Ltd., Shanghai, China) and were identified when similarities greater than 97% were found with the query sequence. The phylogenetic tree of this strain was constructed using the neighbor joining method (1,000 replicates) using the software MEGA-X.

### Pure Culture Fermentation of Sour Porridge

SZ02 were cultured in rice soup medium (2% w/v glucose and 2% w/v soybean oligopeptide) for three generations. A 15 mL aliquot of thus-activated strain SZ02 (approximately 2.52 × 10^8^ CFU/mL) was incubated into 300 mL of rice soup medium containing 2% w/v glucose at 35°C for 24 h to obtain acidic liquid (SLF group). Raw broomcorn millet was sterilized by ultraviolet radiation at 200–280 nm for 2 h and transferred into the acidic liquid at 35°C for 48 h for fermentation. The fermented and unfermented groups were designated FER and NA, respectively.

### Preparation of Rice Flour

Fermented broomcorn millet was deacidified by multiple rinses in distilled water and unfermented broomcorn millet was cleaned by washing and draining, oven-dried at 35°C for 24 h, ground into flour using a blender, and then passed through a 100-mesh stainless steel sieve (0.150 mm).

### Determination of Chemical Composition, pH, and Titratable Acidity

Total starch, crude protein, crude lipid, ash, and the apparent amylose content (AAC) of broomcorn millet was determined according to the methods described by Park et al. (2020). The pH of the supernatant was determined using a pH Meter (Shanghai Lei Magnetic Instrument Co., Ltd., Shanghai, China). Titratable acidity (TA) was determined by titrating with 0.1 mol/L NaOH and expressed as °T.

### Analysis of Pasting Properties

The method of preparation to analyze the pasting properties involved adding 3 g broomcorn millet flour and 25 mL distilled water into the RVA test tank and stirring until there is no agglomeration. The machine speed used was 960 r/min in the first 10 s, and then it was changed to 160 r/min. The test heating program included the following steps: it was held at 50°C for 1 min, raised at 12°C/min to 95°C, held at 95°C for 3.5 min, then cooled to 50°C at 12°C/min, and held again at 50°C for 2 min. The pasting properties of broomcorn millet flour were measured using a Rapid Visco Analyzer (Newport Scientific, Inc. Australia) according to the AACC Method 76-21 (2000).

### Determination of Cooking Quality

Cooking quality of broomcorn millet was determined by analysis of four parameters: iodine blue value (IBV), water absorption rate (WAR), volume expansion ratio (VER), and dry matter of rice soup (DMRS). The specific experimental process refers to Li (2006).

#### Determination of IBV

Rice flour (1 g) was dissolved in 25 mL distilled water in an 80°C water bath for 20 min. The sample was mixed and centrifuged at 6,100 *g* for at least 5 min at 4°C, and the pellet was discarded. To 1 mL of supernatant, 0.5 mL of 0.1 M HCl and 0.5 mL of 0.2% w/v iodine were added, and the volume of the mixture was made up to 10 mL with distilled water. The absorbance was measured at 620 nm in a spectrophotometer (Shanghai Hujing Medical Device Co., Ltd.) after standing for 15 min.

#### Determination of WAR and VER

Ten grams of broomcorn millet was cleaned by washing and draining, and the dried broomcorn millet was placed individually on a wire cage of known weight, rinsed five times with distilled water, and then placed in a beaker. Next, 120 mL distilled water (50°C) was added, and the mixture was placed in a boiling water bath for 10 min. The water was removed, and the remaining mixture was drained and weighed and the volume of grains was measured after placing on the gauze for 30 min.

#### Determination of DMRS

Rice soup produced from cooking raw rice (10 g) was filtered and brought to a final volume of 100 mL with distilled water. The sample were centrifuged at 6600 *g* for at least 10 min at 4°C. After centrifugation, 10 mL of the mixture was removed and placed in an aluminum box of known weight, dried at 100°C for 6 h, and weighed.

#### Calculation of WAR, VER, and DMRS

The following formulae were used to calculate WAR, VER, and DMRS:

$$WAR\left(\%\right)=\frac{A}{B}\times 100\%$$

$$VER\left(\%\right)=\frac{C}{D}\times 100\%$$

$$DMRS\left(mg/g\right)=\frac{E}{10}\times \frac{100}{10}$$

where: A, weight of cooked rice, g; B, weight of raw rice, g; C, volume of cooked rice, mL; D, volume of raw rice, mL; and E, weight of solubilized dry matter, mg.

### Statistical Analysis

All experimental data are presented as means ± standard deviation (SD). Significant differences between different groups are evaluated by one-way analysis of variance (ANOVA). Linear discriminant analysis (LDA) effect size (LEfSe) analysis was used to find the difference species (Biomarker) between each group; the specific operation was as follows: firstly, biomarker with significant difference in abundance between each group was evaluated through Kruskal–Wallis test and Wilcoxon rank-sum test, and then according to LDA score to evaluate the impact of the species on the difference between groups. LEfSe analysis performed using the Python LEfSe package/R package. Abundance of biomarker was determined from heatmaps based on each taxonomy level using R package.

## Results

### ASV Clustering and Annotation

**FIGURE 1:**
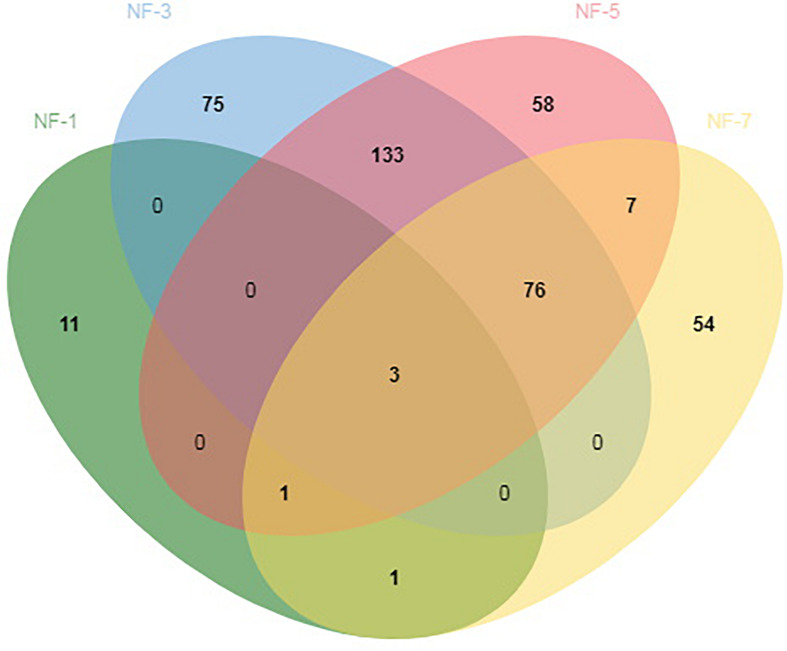
Analysis of amplicon sequence variants (ASVs) shared by different groups. Numbers below groups indicate the number of ASVs within each sector. The number of species in NF-1 is 15; the number of species in NF-3 is 287; the number of species in NF-5 is 278; the number of species in NF-7 is 142; the number of species shared between NF-1 and NF-3 is 3; the number of species shared between NF-1 and NF-5 is 3; the number of species shared between NF-1 and NF-7 is 5; the number of species shared between NF-3 and NF-5 is 212; the number of species shared between NF-3 and NF-7 is 79; the number of species shared between NF-5 and NF-7 is 87; the total shared richness is 3; and the total richness of all the groups is 419.

The high-quality sequence obtained by the DADA2 method from QIIME2 were deduplicated to obtain ASVs. We identified 419 ASVs throughout the fermentation stage. The lowest number of ASVs was found in NF-1 group Only 15, 287 ASVs were found in the NF-3 group, 278 ASVs were found in the NF-5 group, and 142 ASVs were found in the NF-7 group. NF-1, NF-3, NF-5, and NF-7 had 11, 75, 58, and 54 unique ASVs, respectively. In addition, 3 ASVs were detected in all groups (Figure 1).

**FIGURE 2:**
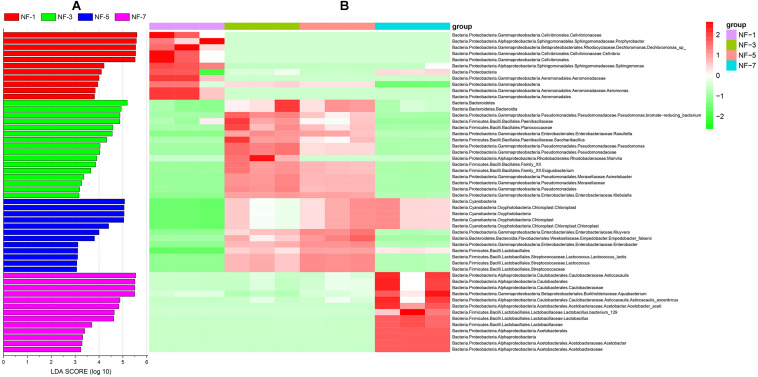
Analysis of sour porridge biomarker by LEfSe analysis based on each taxonomy level. **(A)** Histogram of LDA values (log 10) higher than 3; **(B)** heatmap of the relative abundances of different biomarker based on LEfSe analysis and related taxonomic information at different levels.

LEfSe analysis was used to obtain insights into biomarker based on each taxonomy level at different fermentation stages. LDA scores (log 10 value, greater than 3) revealed that the relative abundance of 52 biomarkers increased significantly throughout the fermentation stage; these included 11 biomarkers in group NF-1, 16 biomarkers in group NF-3, 12 biomarkers in group NF-5, and 13 biomarkers in group NF-7 (Figures 2A,B). *Aeromonas* was significantly enriched in group NF-1. *Klebsiella, Acinetobacter*, and *Pseudomonas* were significantly enriched in group NF-3. The relative abundance of *Enterobacter* and *Kluyvera* were markedly increased in group NF-5. Furthermore, a significant increase in *Acetobacter* and *Lactobacillus* relative abundance were observed in group NF-7.

**FIGURE 3:**
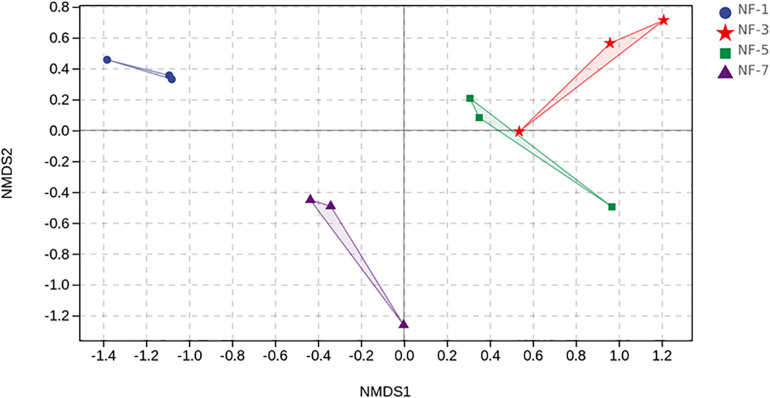
NMDS ordination. NMDS plots demonstrate that sour porridge in different fermentation periods are harboring different bacterial composition.

Table 1 shows the alpha diversity indices of each group. The Chao 1 estimator of group NF-3 was significantly higher (*P* > 0.05) than that of the other groups. The Shannon and Simpson estimators of group NF-3 and group NF-5 were significantly higher than those of the other two groups (*P* < 0.05), while there were no significant differences between groups NF-3 and NF-5 (*P* > 0.05). Cell numbers typically increased, followed by a decrease as the fermentation progressed, and the maximum diversity was found in group NF-3. The beta diversity of the microorganisms in sour porridge, determined by NMDS analysis, showed that groups NF-3 and NF-5 are similar in microbial composition and well separated from the other two groups (Figure 3).

**TABLE 1:** Sour porridge microbiota diversity indices.

Sample	Chao	Shannon	Simpson
NF-1	64.80 ± 14.73^d^	1.33 ± 0.43^c^	0.444 ± 0.172^c^
NF-3	709.98 ± 17.65^a^	5.14 ± 0.05^a^	0.925 ± 0.004^a^
NF-5	663.34 ± 30.29^b^	4.95 ± 0.03^a^	0.907 ± 0.001^a^
NF-7	337.51 ± 12.84^c^	3.24 ± 0.17^b^	0.698 ± 0.031^b^

*Means within a column followed by different letters are significantly different (P < 0.05).*

### Metabolic Pathways of Microbiota in Sour Porridge

**FIGURE 4:**
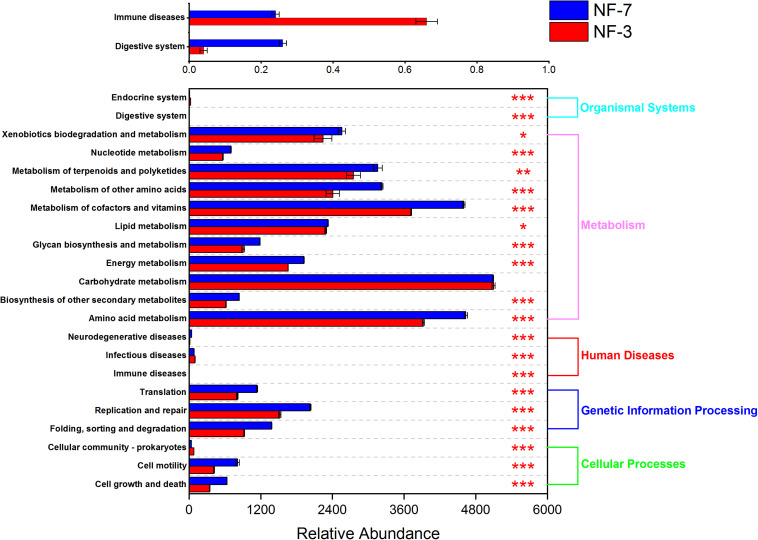
Relative abundance of metabolic pathways at KEGG level 2. Abundance and differences in the predicted functional metagenomes of the sour porridge microbiota. Comparison of the functional pathways of microbes in the NF-3 group and NF-7 group at KEGG level 2. *P < 0.05; **P < 0.01; ***P < 0.001.

**FIGURE 5:**
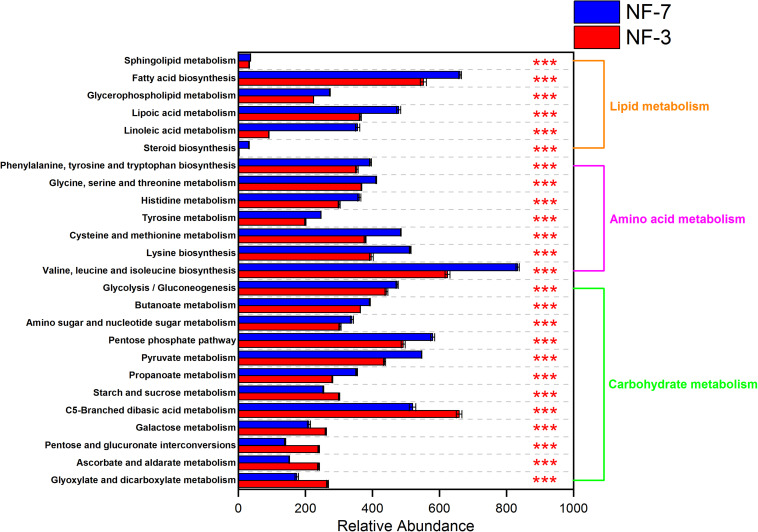
Relative abundance of metabolic pathways at KEGG level 3. Abundance and differences in the predicted functional metagenomes of the sour porridge microbiota. Comparison of the functional pathways of microbes in the NF-3 and NF-7 groups at KEGG level 3. ***P < 0.001.

The functional profiles of the microbial composition at different fermentation stages were analyzed using PICRUSt2 based on the KEGG database. The microbiota in group NF-7 exhibited higher relative abundance of pathways related to metabolism of various substances than those in group NF-3 (*P* < 0.05). However, the relative abundance of carbohydrate metabolic pathways in group NF-7 was slightly lower than that in group NF-3, but the difference was insignificant (*P* > 0.05) (Figure 4). A further 25 pathways related to carbohydrate, lipid, and amino acid metabolism showed significant differences in relative abundance between groups NF-7 and NF-3 (Figure 5). The abundance of six pathways related to lipid metabolism and seven pathways related to amino acid metabolism was significantly higher in group NF-7 than in group NF-3 (*P* < 0.001). The abundance of pathways related to essential amino acid metabolism and biosynthesis, linoleic acid metabolism, lipoic acid metabolism, and fatty acid biosynthesis, which may contribute to the production of various amino acids and short-chain fatty acids in the porridge, was significantly increased in group NF-7. The relative abundances of 12 pathways related to carbohydrate metabolism significantly differed between groups NF-7 and NF-3, with six downregulated and six upregulated pathways. Pentose phosphate pathway, glycolysis/gluconeogenesis and fructose and mannose metabolism were significantly enriched in group NF-7. However, the abundance of pathways related to galactose, starch, and sucrose metabolism was significantly decreased in group NF-7.

### Analysis of Microbial Composition of Sour Porridge

**FIGURE 6:**
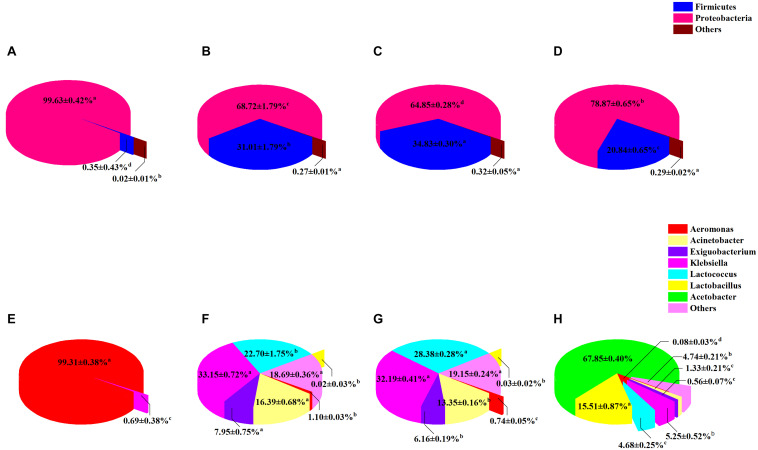
Distribution of sour porridge microbiota composition of different fermentation periods at the phylum **(A)** NF-1; **(B)** NF-3; **(C)** NF-5; and **(D)** NF-7, and genus **(E)** NF-1; **(F)** NF-3; **(G)** NF-5; and **(H)** NF-7 level. The proportions of each phylum in NF-1, NF-3, NF-5, and NF-7 groups are as follows: Firmicutes: 0.35 ± 0.43, 31.01 ± 1.79, 34.83 ± 0.30, and 20.84 ± 0.65%; Proteobacteria: 99.63 ± 0.42, 68.72 ± 1.79, 64.85 ± 0.28, and 78.87 ± 0.65%; others: 0.02 ± 0.01, 0.27 ± 0.01, 0.32 ± 0.05, and 0.29 ± 0.02%. The proportions of each genus in NF-1, NF-3, NF-5, and NF-7 groups are as follows: NF-1 group: Aeromonas: 99.31 ± 0.38%; other: 0.69 ± 0.38%. NF-3 group: Aeromonas: 1.10 ± 0.03%; Acinetobacter: 16.39 ± 0.68%; Exiguobacterium: 7.95 ± 0.75%; Klebsiella: 33.15 ± 0.72%; Lactococcus: 22.70 ± 1.75%; *Lactobacillus*: 0.02 ± 0.03%; other: 18.69 ± 0.36%. NF-5 group: Aeromonas: 0.74 ± 0.05%; Acinetobacter: 13.35 ± 0.16%; Exiguobacterium: 6.16 ± 0.19%: Klebsiella: 32.19 ± 0.41%; Lactococcus: 28.38 ± 0.28%; *Lactobacillus*: 0.03 ± 0.03%: other: 19.15 ± 0.24%. NF-7 group: Aeromonas: 0.08 ± 0.03%; Acinetobacter: 1.33 ± 0.21%; Exiguobacterium: 0.56 ± 0.07%; Klebsiella: 5.25 ± 0.52%: Lactococcus: 4.68 ± 0.25%; *Lactobacillus*: 15.51 ± 0.87%; Acetobacter: 67.85 ± 0.04%; other: 4.74 ± 0.21%.

The composition of sour porridge microbiota at different fermentation periods was analyzed at the phylum and genus levels. Firmicutes and Proteobacteria were the most abundant phyla in all groups. The different groups showed a statistically significant difference in microbial profile at the phylum level (*P* < 0.05) (Figures 6A–D). Proteobacteria (99.63%) was the most abundant phylum on the first day of fermentation (group NF-1). Firmicutes (20.84–34.83%) and Proteobacteria (64.85–78.87%) were the dominant phyla on days 3–7. The abundance of Firmicutes gradually increased as the fermentation progressed, then significantly decreased to 20.84% (*P* < 0.05) on day 7 (group NF-7). The genus-level microbial profile of sour porridge during different fermentation stages revealed that *Aeromonas* (99.31%) was the dominant genus in group NF-1 (Figures 6E–H), but its abundance gradually decreased as the fermentation progressed and dropped significantly to 0.08% (*P* < 0.05) in group NF-7. *Acinetobacter*, *Klebsiella*, and *Exiguobacterium* were detected in group NF-3, accounting for 16.39, 33.15, and 7.95% of the total microbiota, respectively, and as the fermentation progressed, the abundance decreased gradually, dropping to 1.33, 5.25, and 0.56%, respectively, in group NF-7 (*P* < 0.05). *Lactococcus* was found in group NF-3, accounting for 22.70% of the total bacteria; its abundance markedly increased to 28.38% in group NF-5 (*P* < 0.05) but decreased significantly to 4.68% in group NF-7 (*P* < 0.05). Notably, the abundance of *Lactobacillus* (0–0.03%) and *Acetobacter* (0%) was extremely low on days 1–5, but increased markedly to 15.51 and 67.85%, respectively, on day 7 (*P* < 0.05). This increase may be due to the gradually decrease in sour porridge pH caused by organic acids produced during fermentation (Supplementary Figure 2), which favors the growth of acid-resistant *Lactobacillus* and *Acetobacter* genus (Yang et al., 2020). However, *Lactococcus* exhibits poorer acid resistance than *Lacticaseibacillus*, which causes *Lactococcus* to gradually lose its competitive edge (Savoie et al., 2007). Additionally, *Lactobacillus* may produce bacteriocin, organic acids, and antimicrobial substances to suppress the growth of pathogenic microorganisms (Guan et al., 2020). Since the above-mentioned bacteria are not acid-tolerant, Additionally, the bacteriocin produced by lactic acid bacteria may inhibit the growth of the bacteria; this might be responsible for the changes in the relative abundance of *Acinetobacter*, *Klebsiella*, and *Exiguobacterium*.

### Isolation and Identification of Rapid Acid-Producing Strains

**FIGURE 7:**
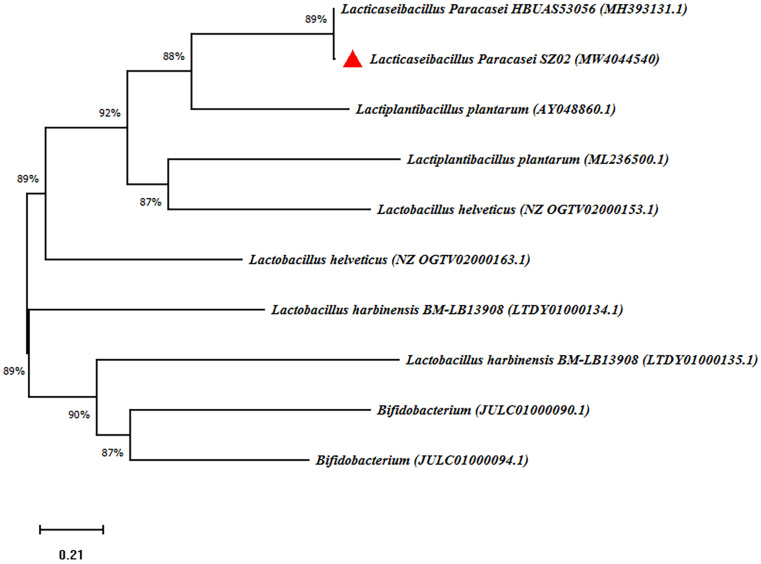
Phylogenetic relationship of SZ02 with related species based on partial 16S rDNA gene sequence analysis. The phylogenetic tree was constructed using the neighbor-joining method (MEGA-X). The numbers at the nodes are bootstrap confidence levels (percentage) from 1,000 replicates. The scale bar represents 0.05 substitutions per nucleotide position. Reference sequences were obtained from the GenBank nucleotide sequence database.

We isolated 40 strains with Gram-positive and catalase-negative properties in rice soup solid medium plate containing 2% w/v glucose, 2% w/v agar, and 2% w/v soybean oligopeptide. Seven strains acidified the medium to pH 4.35 or lower. Strains SZ01, SZ02, and SZ03 produce relatively large quantities of acid, evidenced by the low pH and quantitatively determined by titration of the fermentation liquid (Supplementary Table 1). SZ02 was selected for pure culture fermentation experiments following comprehensively consideration of the pH and TA of rice soup. Full-length 16S rRNA gene sequencing identified SZ02 strain as a unique strain of *Lacticaseibacillus paracasei* (accession number: MW404454); its relationship to other species is shown in the phylogenetic tree (Figure 7).

### Analysis of pH, Titratable Acidity, and Chemical Composition

Acidic liquid was prepared by inoculating strain SZ02 into rice soup, and raw rice was added for further fermentation. The initial pH and TA of rice soup was 6.55 and 4.61°T, which changed to 3.94 and 13.85°T (*P* < 0.05) after 24 h inoculation, then to 3.22 and 46.96°T (*P* < 0.05) after 48 h (Table 2). Crude protein and crude fat content of broomcorn millet decreased from 8.17 and 2.27 to 7.23 and 1.46%, respectively (*P* < 0.05). The protein in broomcorn millet is the only nitrogen source for the growth and reproduction of lactic acid bacteria, and the protein content gradually decreased with time (Li et al., 2019). The ash content decreased from 0.30 to 0.21 (*P* < 0.05), and fermentation destroyed the structure of broomcorn millet, releasing minerals into the acid liquid and leading to a decrease in ash content. Interestingly, the content of starch increased from 69.24 to 70.41% after fermentation (*P* < 0.05), suggesting that the consumption of starch in the broomcorn millet was significantly lower than that of other components during fermentation. AAC increased from 15.61 to 17.84% after fermentation (*P* < 0.05).

**TABLE 2:** pH, titratable acidity, and chemical composition of sour porridge.

Sample	Acid liquid		Broomcorn millet				
	pH	TA (°T)	Crude protein	Crude fat	Total starch	Ash	Amylose
NA	6.55 ± 0.01^a^	4.61 ± 0.11^a^	8.17 ± 0.22^a^	2.27 ± 0.18^a^	69.24 ± 0.12^b^	0.30 ± 0.02^*a*^	15.61 ± 0.36^b^
SLF	3.94 ± 0.05^b^	13.85 ± 0.23^b^					
FER	3.22 ± 0.02^c^	46.96 ± 1.39^c^	7.23 ± 0.10^b^	1.46 ± 0.04^b^	70.41 ± 0.05^a^	0.21 ± 0.04^b^	17.84 ± 0.17^a^

*Means within a column followed by different letters are significantly different (P < 0.05).*

### Analysis of the Pasting Properties of Broomcorn Millet

**FIGURE 8:**
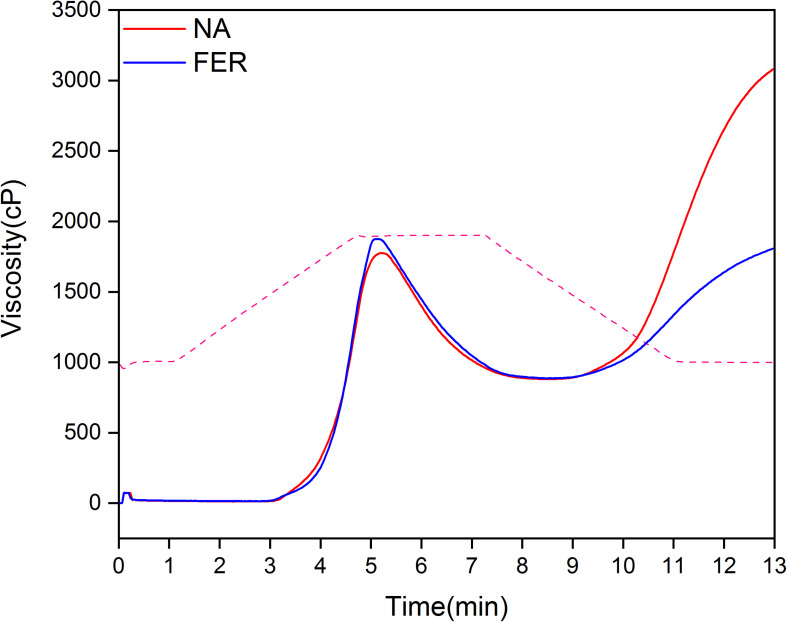
Pasting profile of broomcorn millet flour. Pasting profile of natural rice flour and fermented rice flour. Red and blue solid lines represent the viscosity (cP) profile of natural sample and fermented sample, respectively. The dotted line is the temperature (°C).

After fermentation, the pasting properties of broomcorn millet flour changed significantly, with an increase in pasting viscosity (PV) and breakdown viscosity (BD), and a decrease in final viscosity (FV) and setback viscosity (SB) (*P* < 0.05) (Figure 8 and Table 3).

**TABLE 3:** Pasting properties of broomcorn millet.

Sample	PV	TV	BD	FV	SB
NA	1776.00 ± 25.98^b^	881.00 ± 16.70^a^	895.00 ± 28.62^b^	3079.33 ± 52.04^a^	2198.33 ± 35.53^a^
FER	1878.67 ± 26.50^a^	886.00 ± 24.25^a^	992.67 ± 19.86^a^	1806.33 ± 33.86^b^	920.33 ± 10.50^b^

*Means within a column followed by different letters are significantly different (P < 0.05).*

### Analysis of the Cooking Properties of Broomcorn Millet

The WAR and VER of broomcorn millet increased significantly from 206.32 and 247.67 to 246.57 and 286.66% after fermentation, respectively (*P* < 0.05) (Table 4).

**TABLE 4:** Cooking properties of broomcorn millet.

Sample	DMRS (mg/g)	IBV	VER (%)	WAR (%)
NA	18.26 ± 0.25^b^	0.321 ± 0.004^b^	247.67 ± 3.31^b^	206.32 ± 3.09^b^
FER	20.10 ± 0.22^a^	0.461 ± 0.007^a^	286.66 ± 5.17^a^	246.57 ± 1.94^a^

*Means within a column followed by different letters are significantly different (P < 0.05).*

## Discussion

This study investigated the microbial profile during different stages of fermentation of sour porridge and explored the utility of pure culture fermentation for improving the edible quality of sour porridge.

The microbial profile analysis revealed that the relative abundance of bacteria that secrete toxic substances and cause various infections and diseases markedly reduced in the post-fermentation stage (group NF-7) of broomcorn millet. *Acinetobacter* and *Pseudomonas* are pathogens with high antimicrobial resistance and cause inflammation and a variety of diseases (Fishbain and Peleg, 2010; Ruiz-Roldán et al., 2020). *Klebsiella* and *Enterobacter* belong to the family Enterobacteriaceae and are the most common foodborne pathogens (Fasugba et al., 2015) that can infect the urinary tract, blood, and the gastrointestinal tract (Davin-Regli et al., 2019; Bitew and Tsige, 2020; Wu et al., 2020). The aforementioned pathogenic bacteria were significantly decreased in group NF-7, while non-disease-causing *Lacticaseibacillus*, considered the main contributor to flavor, mouthfeel, and health benefits of sour porridge, and the less harmful *Acetobacter*, were significantly enriched at the same time. This result was consistent with previous reports that *Lactobacillus* and *Acetobacter* are the dominant bacteria in sour porridge (Wang et al., 2018). Notably, the present study demonstrates that it takes at least a week or even longer for enrichment of *Lactobacillus* in sour porridge.

The microorganisms in group NF-7 are predicted to metabolize amino acids, lipids, and other metabolites of broomcorn millet at a faster rate than those in group NF-3. The unique flavor and nutritional value of sour porridge are attributed to the beneficial metabolites produced by microbial metabolic activities, including organic acids, amino acids, short-chain fatty acids, and small molecular substances. Remarkably, pathways of the xenobiotics biodegradation and metabolism were higher in group NF-7 than in group NF-3 (*P* < 0.05). Compared to group NF-3, the potential of group NF-7 microorganisms to metabolize monosaccharides, such as fructose and mannose was significantly increased, and the abundance of Embden-Meyerhof-Parnas (EMP) and pentose phosphate (HMP) pathways also increased significantly. It should be noted that the ability to metabolize starch and sucrose in group NF-7 was dramatically lower than that of group NF-3. This implies that the microorganisms group NF-7 utilize the starch breakdown products from group NF-3. In the pure fermentation experiment, We found a significant increase in AAC, consistent with previous report (Yi et al., 2017). Studies have found that the enzymes or acids produced by fermentation primarily act in the amorphous region containing the branch point of amylopectin, which leads to the breakage of the branching point of amylopectin and increases the amylose content (Blazek and Gilbert, 2010; Gope et al., 2016; Ratnaningsih et al., 2020). In addition, some long branch-chains of amylopectin can bind with iodine to increase the ACC (Luo et al., 2020), but this phenomenon will be limited when it forms complexes with lipids (Valdez-Arana et al., 2020; Kim et al., 2020).

The pasting properties of starch-based foods affects their edible quality and are closely related to the average chain length of amylose and amylopectin, amylose/amylopectin ratio; protein and lipid content of starch granules; and average molecular weight (Lin et al., 2012; Ai et al., 2013; Majzoobi et al., 2014). Fermentation affects the pasting characteristics of rice flour or starch from different sources (Putri et al., 2012; Li et al., 2015; Surojanametakul et al., 2019). The pasting properties of broomcorn millet (PV, BD, FV, and SB) were significantly changed following fermentation. PV is the maximum viscosity of rice flour during heating, which affects the final quality of starch-based food (Thomas and Atwell, 1999). The PV is associated with the swelling power of starch and is affected by amylose chain length and amylopectin chain length distribution. Amylopectin short chains promote the expansion of starch, and amylose acts as a diluent. However, when the amylose and amylopectin long chains form a complex with lipid, they seriously affect the water absorption and expansion of starch granules (Tester and Morrison, 1990; Wang R. et al., 2019). In addition, the protein adheres to starch and affects the water absorption and swelling of starch granules (Sandhu et al., 2018) and PV of wheat starch increases after protein removal (Li et al., 2016). The increase in PV observed in this study may be due to the increase of short-chain amylopectin content and the degradation of proteins and lipids in broomcorn millet related to starch granules losing their bonding with water molecules. BD, which reflects the thermal stability of starch granules, also increased significantly after fermentation, indicating that fermentation weakens the interaction between proteins, lipids, and starch in broomcorn millet. Starch granules are more likely to swell and rupture under shearing and heating stress (Zhang et al., 2019; Zhu et al., 2020), releasing polysome and amylose and increasing the viscosity. When starch pastes start to cool down there is rearrangement of the solubilized starch molecules, especially solubilized amylose, which results in increased FV. This process is called retrogradation, which induces adverse effects on starch-based food nutritional values and sensory qualities. Many studies have demonstrated that the amylose content is positively correlated with SB (Li et al., 2020; Soleimanpour et al., 2020). In our experiments, we found that amylose content increased after fermentation, but the SB value decreased significantly (*P* < 0.05). We suggest this may be due to the organic acids and protein residues produced by fermentation hindering the rearrangement of starch molecules (Park et al., 2020). In addition, fermentation may reduce molecular weights of starch, and produce more amylose and amylopectin molecules with a lower degree of polymerization. This may hinder the formation of hydrogen bonds among starch molecules, thereby inhibiting short-term retrogradation of starch (Zuo et al., 2017).

Water absorption rate reflects the hydration ability of broomcorn millet and affects the VER (Shen et al., 2019). The improvement in WAR and VER following fermentation may be due to the destruction of some hydrophobic amino acid groups and the increase of short-chain amylopectin after fermentation. Starch forms starch-lipid complexes during the pasting process (Castellanos-Gallo et al., 2019), and these complexes inhibit the starch granule swelling in water (Wang H. et al., 2019). The stability of the complex is affected by the length and saturation level of lipid chains (Chen et al., 2018). Fermentation produces short-chain fatty acids and amylose, reducing the interaction between amylose chain and fatty acid chain as well as the complex stability, thereby promoting starch granule swelling in water. In addition, the surface of fermented broomcorn millet starch granules have more holes and cracks compared with unfermented broomcorn millet (Supplementary Figures 3A,B), which may be explained by the degradation of starch granules induced by organic acids and/or amylase produced by lactic acid bacteria (Majzoobi and Beparva, 2014; Li et al., 2015). Huber and BeMiller (1997) confirmed that there are many small pores on the surface of starch granules, which are large enough to allow organic acids and enzymes to enter the interior of the granules and create additional holes and cracks. This may promote the water absorption and volume expansion of broomcorn millet. With the increase of VER, amylose content and the abundance macromolecular substances are easier to overflow by heating (Elkhalifa et al., 2017), which leads to a significant increase in DMRS (from 18.26 to 20.10) and IBV (form 0.321 to 0.461) of the fermented broomcorn millet (*P* < 0.05). Previous studies showed that high DMRS and IBV values indicates a high palatability of porridge (Zhan et al., 2007; He and Chen, 2009). Altogether, this study shows that sour porridge made from the fermented broomcorn millet has a better taste and edible quality compared with unfermented millet.

## Conclusion

The microbial composition and diversity of sour porridge at different fermentation periods are significantly different. *Aeromonas*, *Acinetobacter*, and *Klebsiella* were the dominant genus in the pre-fermentation stage and *Lactobacillus* and *Acetobacter* were the main dominant bacteria in the post-fermentation stage. Metabolic function prediction revealed that group NF-7 microorganisms had increased metabolism of amino acids, lipids, and monosaccharides, while their metabolism of starch and sucrose was weaker compared with group NF-3.

Since the microbiota in natural fermentation is dependent on the environment, it is prone to unfavorable conditions leading to inconsistent product quality. Therefore, a controlled fermentation experiments using *Lacticaseibacillus paracasei* strain SZ02 was performed to determine its efficiency and edibility compared with unfermented culture. Fermentation of the pure culture increased the water absorption and volume expansion rate of broomcorn millet, and promoted the dissolution of solids and amylose, thereby improving the edible quality of sour porridge. In addition, fermentation increased the PV, reduced the SB of the broomcorn millet, and inhibited the negative effects of starch aging behaviors on the edible quality of sour porridge. This suggests *Lacticaseibacillus paracasei* strain SZ02 has the potential to be the primary organism in the industrialized production of sour porridge.

## Data Availability Statement

The datasets presented in this study can be found in online repositories. The names of the repository/repositories and accession number(s) can be found below: https://www.ncbi.nlm.nih.gov/genbank/, MW404454 and https://www.ncbi.nlm.nih.gov/, PRJNA687736.

## Author Contributions

YX and LZ designed the research study. CW, YX, AX, and BY performed all experiments and wrote the manuscript. CW, YX, LZ, YS, HZ, and HG conducted the experiments and data analysis. All authors read and approved the manuscript.

## Conflict of Interest

The authors declare that the research was conducted in the absence of any commercial or financial relationships that could be construed as a potential conflict of interest.

## Publisher’s Note

All claims expressed in this article are solely those of the authors and do not necessarily represent those of their affiliated organizations, or those of the publisher, the editors and the reviewers. Any product that may be evaluated in this article, or claim that may be made by its manufacturer, is not guaranteed or endorsed by the publisher.

## Supplementary Material

Supplementary Figure 1 Production of sour porridge by natural fermentation.

Supplementary Figure 2 pH of sour porridge. pH change of sour porridge during 1–7 days of fermentation.

Supplementary Figure 3 Scanning electron micrograph of natural and fermented starch granules. **(A)** Natural starch granules (10,000×). **(B)** Fermented starch granules (10,000×).

Supplementary Table 1 Acid producing and morphological properties of selected lactic acid bacteria.

## References

[B1] Ai Y. Hasjim J. Jane J. (2013). Effects of lipids on enzymatic hydrolysis and physical properties of starch. *Carbohydr. Polym.* 92 120–127. 10.1016/j.carbpol.2012.08.092 23218274

[B2] Amadou I. Gounga M. E. Shi Y.-H. Le G.-W. (2014). Fermentation and heat-moisture treatment induced changes on the physicochemical properties of foxtail millet (Setaria italica) flour. *Food Bioprod. Process.* 92 38–45. 10.1016/j.fbp.2013.07.009

[B3] Bitew A. Tsige E. (2020). High prevalence of multidrug-resistant and extended-spectrum β-lactamase-producing *Enterobacteriaceae*: a cross-sectional study at Arsho Advanced Medical Laboratory, Addis Ababa, Ethiopia. *J. Trop. Med.* 2020:6167234. 10.1155/2020/6167234 32411256 PMC7210541

[B4] Blazek J. Gilbert E. P. (2010). Effect of enzymatic hydrolysis on native starch granule structure. *Biomacromolecules* 11 3275–3289. 10.1021/bm101124t 21033657

[B5] Çabuk B. Stone A. K. Korber D. R. Tanaka T. Nickerson M. T. (2018). Effect of *Lactobacillus plantarum* fermentation on the surface and functional properties of pea protein-enriched flour. *Food Technol. Biotechnol.* 56 411–420. 10.17113/ftb.56.03.18.5449 30510484 PMC6233015

[B6] Castellanos-Gallo L. Galicia-García T. Estrada-Moreno I. Mendoza-Duarte M. E. Márquez-Meléndez R. Márquez-Goméz M. (2019). Development of an expanded snack of rice starch enriched with amaranth by extrusion process. *Molecules* 24:2430. 10.3390/molecules24132430 31269663 PMC6651392

[B7] Chen B. Jia X. Miao S. Zeng S. Guo Z. Zhang Y. (2018). Slowly digestible properties of lotus seed starch-glycerine monostearin complexes formed by high pressure homogenization. *Food Chem.* 252 115–125. 10.1016/j.foodchem.2018.01.054 29478521

[B8] Chen Z.-J. Yang X.-Q. Wu N. Dai L.-J. Li C.-G. (2002). Isolation and study of biological properties of lactic acid bacteria from acidic-gruel from Hetao area Inner Mongolia. *J. Inner Mongolia Agric. Univ.* 23 62–65.

[B9] Danczak R. E. Chu R. K. Fansler S. J. Goldman A. E. Graham E. B. Tfaily M. M. (2020). Using metacommunity ecology to understand environmental metabolomes. *Nat. Commun.* 11:6369. 10.1038/s41467-020-19989-y 33311510 PMC7732844

[B10] Davin-Regli A. Lavigne J.-P. Pagès J.-M. (2019). *Enterobacter* spp.: update on taxonomy, clinical aspects, and emerging antimicrobial resistance. *Clin. Microbiol. Rev.* 32 e00002–e19. 10.1128/CMR.00002-19 31315895 PMC6750132

[B11] Elkhalifa A. E. O. Bernhardt R. Cardone G. Marti A. Iametti S. Marengo M. (2017). Physicochemical properties of sorghum flour are selectively modified by combined germination-fermentation. *J. Food Sci. Technol.* 54 3307–3313. 10.1007/s13197-017-2781-7 28974816 PMC5602995

[B12] Fasugba O. Gardner A. Mitchell B. G. Mnatzaganian G. (2015). Ciprofloxacin resistance in community- and hospital-acquired *Escherichia coli* urinary tract infections: a systematic review and meta-analysis of observational studies. *BMC Infect. Dis.* 15:545. 10.1186/s12879-015-1282-4 26607324 PMC4660780

[B13] Fishbain J. Peleg A. Y. (2010). Treatment of *Acinetobacter* infections. *Clin. Infect. Dis.* 51 79–84. 10.1086/653120 20504234

[B14] Gope S. Samyor D. Paul A. K. Das A. B. (2016). Effect of alcohol-acid modification on physicochemical, rheological and morphological properties of glutinous rice starch. *Int. J. Biol. Macromol.* 93 860–867. 10.1016/j.ijbiomac.2016.09.057 27645925

[B15] Guan C. Tao Z. Wang L. Zhao R. Chen X. Huang X. (2020). Isolation of novel *Lactobacillus* with lipolytic activity from the vinasse and their preliminary potential using as probiotics. *AMB Express* 10:91. 10.1186/s13568-020-01026-2 32415368 PMC7229107

[B16] He J. F. Chen Z. G. (2009). Evaluation of rice quality of different varieties. *Fujian Sci. Technol. Rice Wheat* 27 31–33.

[B17] Huber K. C. BeMiller J. N. (1997). Visualization of channels and cavities of corn and sorghum starch granules. *Cereal Chem.* 74 537–541. 10.1094/CCHEM.1997.74.5.537

[B18] Kim H. R. Choi S. J. Choi H. D. Park C. S. Moon T. W. (2020). Amylosucrase-modified waxy potato starches recrystallized with amylose: the role of amylopectin chain length in formation of low-digestible fractions. *Food Chem.* 318:126490. 10.1016/j.foodchem.2020.126490 32146307

[B19] Li C. Wu A. Yu W. Hu Y. Li E. Zhang C. (2020). Parameterizing starch chain-length distributions for structure-property relations. *Carbohydr. Polym.* 241:116390. 10.1016/j.carbpol.2020.116390 32507172

[B20] Li S. C. Lin H. P. Chang J. S. Shih C. K. (2019). *Lactobacillus acidophilus*-fermented germinated brown rice suppresses preneoplastic lesions of the colon in rats. *Nutrients* 11:2718. 10.3390/nu11112718 31717536 PMC6893647

[B21] Li T.-Z. (2006). Research on the effect of bran removal degree on rice. *Food Sci. Technol.* 3 96–100.

[B22] Li W. Wu G. Luo Q. Jiang H. Zheng J. Ouyang S. (2016). Effects of removal of surface proteins on physicochemical and structural properties of A- and B-starch isolated from normal and waxy wheat. *J. Food Sci. Technol.* 53 2673–2685. 10.1007/s13197-016-2239-3 27478223 PMC4951420

[B23] Li X. Wang C. Lu F. Zhang L. Yang Q. Mu J. (2015). Physicochemical properties of corn starch isolated by acid liquid and l-cysteine. *Food Hydrocoll.* 44 353–359. 10.1016/j.foodhyd.2014.09.003

[B24] Liao L. Wu W. (2017). Fermentation effect on the properties of sweet potato starch and its noodle’s quality by *Lactobacillus plantarum*: fermentation starch for noodles. *J. Food Process. Eng.* 40:e12460. 10.1111/jfpe.12460

[B25] Lin J.-H. Pan C.-L. Singh H. Chang Y.-H. (2012). Influence of molecular structural characteristics on pasting and thermal properties of acid-methanol-treated rice starches. *Food Hydrocoll.* 26 441–447. 10.1016/j.foodhyd.2010.11.016

[B26] Lu Z.-H. Li L.-T. Cao W. Li Z.-G. Tatsumi E. (2003). Influence of natural fermentation on physico-chemical characteristics of rice noodles. *Int. J. Food Sci. Tech.* 38 505–510. 10.1046/j.1365-2621.2003.00701.x

[B27] Luo J. Li Z. Gidley M. J. Bird A. R. Tetlow I. J. Fitzgerald M. (2020). Functional genomic validation of the roles of soluble starch synthase IIa in Japonica rice endosperm. *Front. Genet.* 11:289. 10.3389/fgene.2020.00289 32300357 PMC7142255

[B28] Majzoobi M. Beparva P. (2014). Effects of acetic acid and lactic acid on physicochemical characteristics of native and cross-linked wheat starches. *Food Chem.* 147 312–317. 10.1016/j.foodchem.2013.09.148 24206724

[B29] Majzoobi M. Beparva P. Farahnaky A. Badii F. (2014). Effects of malic acid and citric acid on the functional properties of native and cross-linked wheat starches. *Starch Stärke* 66 491–495. 10.1002/star.201300188

[B30] Melini F. Melini V. Luziatelli F. Ficca A. G. Ruzzi M. (2019). Health-promoting components in fermented foods: an up-to-date systematic review. *Nutrients* 11:1189. 10.3390/nu11051189 31137859 PMC6567126

[B31] Park J. Sung J. M. Choi Y.-S. Park J.-D. (2020). Effect of natural fermentation on milled rice grains: physicochemical and functional properties of rice flour. *Food Hydrocoll.* 108:106005. 10.1016/j.foodhyd.2020.106005

[B32] Putri W. D. R. Haryadi, Marseno D. W. Cahyanto M. N. (2012). Role of lactic acid bacteria on structural and physicochemical properties of sour cassava starch. *APCBEE Procedia* 2 104–109. 10.1016/j.apcbee.2012.06.019

[B33] Ratnaningsih N. Suparmo H. E. Harmayani E. Marsono Y. (2020). Physicochemical properties, in vitro starch digestibility, and estimated glycemic index of resistant starch from cowpea (Vigna unguiculata) starch by autoclaving-cooling cycles. *Int. J. Biol. Macromol.* 142 191–200. 10.1016/j.ijbiomac.2019.09.092 31521656

[B34] Ruiz-Roldán L. Rojo-Bezares B. de Toro M. López M. Toledano P. Lozano C. (2020). Antimicrobial resistance and virulence of *Pseudomonas* spp. among healthy animals: concern about exolysin ExlA detection. *Sci. Rep.* 10:11667. 10.1038/s41598-020-68575-1 32669597 PMC7363818

[B35] Sandhu R. S. Singh N. Kaler R. S. S. Kaur A. Shevkani K. (2018). Effect of degree of milling on physicochemical, structural, pasting and cooking properties of short and long grain Indica rice cultivars. *Food Chem.* 260 231–238. 10.1016/j.foodchem.2018.03.092 29699667

[B36] Savoie S. Champagne C. P. Chiasson S. Audet P. (2007). Media and process parameters affecting the growth, strain ratios and specific acidifying activities of a mixed lactic starter containing aroma-producing and probiotic strains. *Appl. Microbiol.* 103 163–174. 10.1111/j.1365-2672.2006.03219.x 17584462

[B37] Shen L. Zhu Y. Wang L. Liu C. Liu C. Zheng X. (2019). Improvement of cooking quality of germinated brown rice attributed to the fissures caused by microwave drying. *J. Food Sci. Technol.* 56 2737–2749. 10.1007/s13197-019-03765-y 31168155 PMC6525723

[B38] Soleimanpour M. Tamaddon A. M. Kadivar M. Abolmaali S. S. Shekarchizadeh H. (2020). Fabrication of nanostructured mesoporous starch encapsulating soy-derived phytoestrogen (genistein) by well-tuned solvent exchange method. *Int. J. Biol. Macromol.* 159 1031–1047. 10.1016/j.ijbiomac.2020.05.124 32439450

[B39] Surojanametakul V. Panthavee W. Satmalee P. Phomkaivon N. Yoshihashi T. (2019). Effect of traditional dried starter culture on morphological, chemical and physicochemical properties of sweet fermented glutinous rice products. *J. Agric. Sci.* 11:43. 10.5539/jas.v11n6p43

[B40] Tester R. F. Morrison W. R. (1990). Swelling and gelatinization of cereal starches. I. Effects of amylopectin, amylose, and lipids. *Cereal Chem.* 67 551–557.

[B41] Thomas D. J. Atwell W. A. (1999). *Gelatinization, Pasting and Retrogradation, Starches.* St Paul, MN: American Association of Cereal Chemists.

[B42] Valdez-Arana J. D. Steffolani M. E. Repo-Carrasco-Valencia R. Pérez G. T. Condezo-Hoyos L. (2020). Physicochemical and functional properties of isolated starch and their correlation with flour from the Andean Peruvian quinoa varieties. *Int. J. Biol. Macromol.* 147 997–1007. 10.1016/j.ijbiomac.2019.10.067 31743707

[B43] Wang H. Wu Y. Wang N. Yang L. Zhou Y. (2019). Effect of water content of high-amylose corn starch and glutinous rice starch combined with lipids on formation of starch-lipid complexes during deep-fat frying. *Food Chem.* 278 515–522. 10.1016/j.foodchem.2018.11.092 30583406

[B44] Wang Q. Liu C. Jing Y. Fan S. Cai J. (2019). Evaluation of fermentation conditions to improve the sensory quality of broomcorn millet sour porridge. *LWT* 104 165–172. 10.1016/j.lwt.2019.01.037

[B45] Wang R. Liu P. Cui B. Kang X. Yu B. (2019). Effects of different treatment methods on properties of potato starch-lauric acid complex and potato starch-based films. *Int. J. Biol. Macromol.* 124 34–40. 10.1016/j.ijbiomac.2018.11.207 30472268

[B46] Wang Y.-R. She M.-N. Liu K.-L. Zhang Z.-D. Quan S. (2018). Diversity of bacteria microflora in acidic-gruel of Inner Mongolia area by MiSeq high throughput sequencing. *Sci. Technol. Food Ind.* 39 124–129.

[B47] Wu W. Feng Y. Zong Z. (2020). Precise species identification for *Enterobacter*: a genome sequence-based study with reporting of two novel species, *Enterobacter quasiroggenkampii* sp. nov. and *Enterobacter quasimori* sp. nov. *mSystems* 5 e00527–20. 10.1128/mSystems.00527-20 32753511 PMC7406230

[B48] Xu J. Chen L. Guo X. Liang Y. Xie F. (2020). Understanding the multi-scale structure and digestibility of different waxy maize starches. *Int. J. Biol. Macromol.* 144 252–258. 10.1016/j.ijbiomac.2019.12.110 31846664

[B49] Yang X. Sun H. Tu L. Jin Y. Zhang Z. Wang M. (2020). Kinetics of enzymatic synthesis of cyanidin-3-glucoside lauryl ester and its physicochemical property and proliferative effect on intestinal probiotics. *Biology (Basel)* 9:205. 10.3390/biology9080205 32759690 PMC7465376

[B50] Yi C. Yang Y. Zhou S. He Y. (2017). Role of lactic acid bacteria in the eating qualities of fermented rice noodles. *Cereal Chem.* 94 349–356. 10.1094/CCHEM-07-16-0187-R

[B51] Yuan M.-L. Lu Z.-H. Cheng Y.-Q. Li L.-T. (2008). Effect of spontaneous fermentation on the physical properties of corn starch and rheological characteristics of corn starch noodle. *J. Food Eng.* 85 12–17. 10.1016/j.jfoodeng.2007.06.019

[B52] Zhan X.-M. Zheng T.-S. Tao J.-H. (2007). Study on application of texture analyzer in quality evaluation of rice. *Food Sci.* 28 62–65.

[B53] Zhang K. Dai Y. Hou H. Li X. Dong H. Wang W. (2019). Influences of grinding on structures and properties of mung bean starch and quality of acetylated starch. *Food Chem.* 294 285–292. 10.1016/j.foodchem.2019.05.055 31126465

[B54] Zhu L. Zhang Y. Wu G. Qi X. Dag D. Kong F. (2020). Characteristics of pasting properties and morphology changes of rice starch and flour under different heating modes. *Int. J. Biol. Macromol.* 149 246–255. 10.1016/j.ijbiomac.2020.01.161 31958556

[B55] Zuo Y. Liu W. Xiao J. Zhao X. Zhu Y. Wu Y. (2017). Preparation and characterization of dialdehyde starch by one-step acid hydrolysis and oxidation. *Int. J. Biol. Macromol.* 103 1257–1264. 10.1016/j.ijbiomac.2017.05.188 28587965

