# Supplementary figures and images for: Analysis of Sour Porridge Microbiota and Improvement of Cooking Quality via Pure Culture Fermentation Using Lacticaseibacillus paracasei Strain SZ02
Source: Front Microbiol. 2021 Aug 26;12:712189. doi: 10.3389/fmicb.2021.712189 (PMC8428527; doi:10.3389/fmicb.2021.712189)

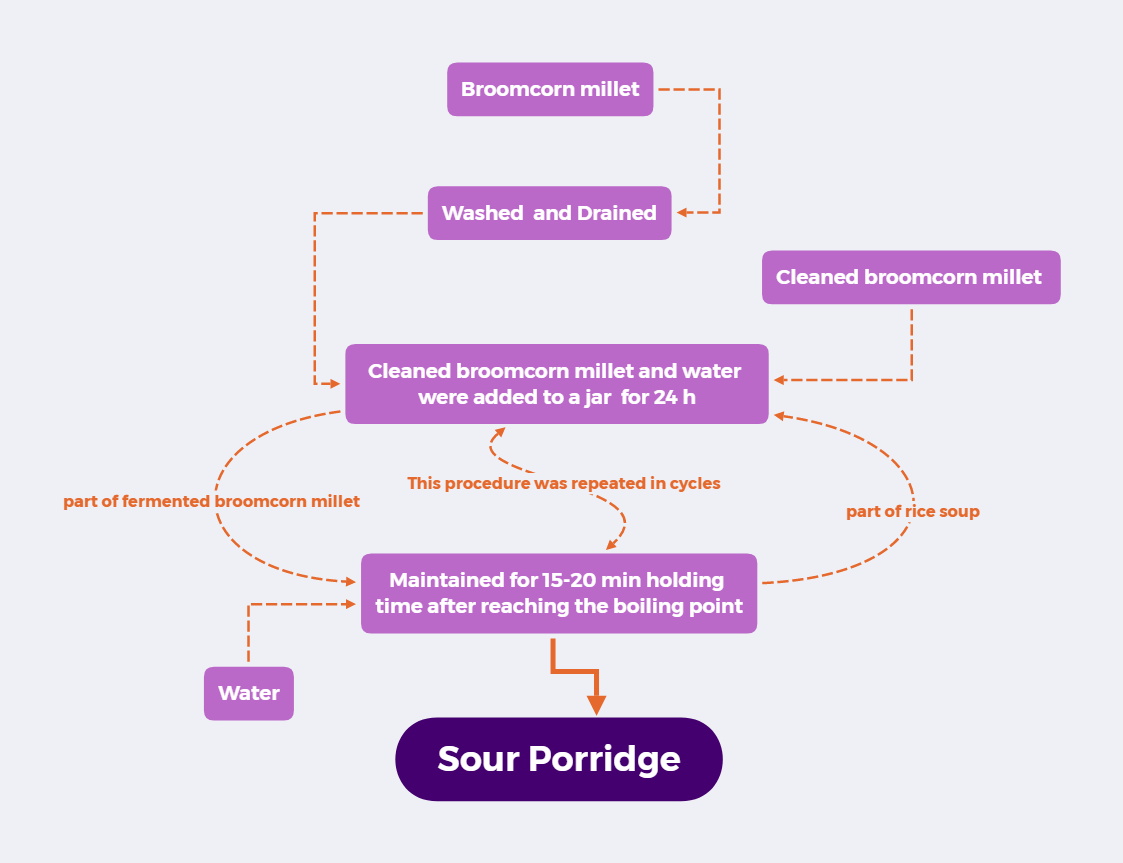

Supplement: Supplementary Figure 1 — Production of sour porridge by natural fermentation. [file Image_1.JPEG]

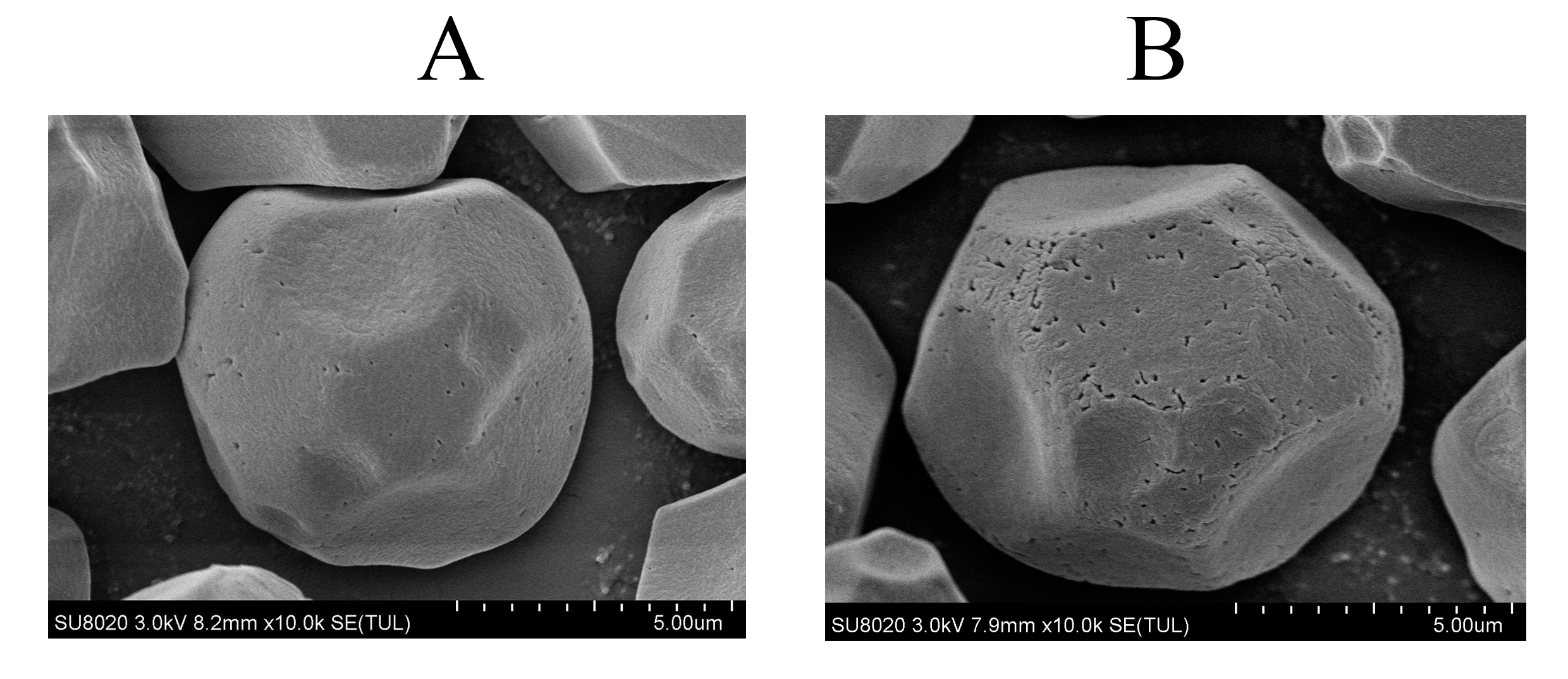

Supplement: Supplementary Figure 3 — Scanning electron micrograph of natural and fermented starch granules. (A) Natural starch granules (10,000×). (B) Fermented starch granules (10,000×). [file Image_3.TIF]
