# Supplementary material for: Analysis of Sour Porridge Microbiota and Improvement of Cooking Quality via Pure Culture Fermentation Using Lacticaseibacillus paracasei Strain SZ02
Source: Front Microbiol. 2021 Aug 26;12:712189. doi: 10.3389/fmicb.2021.712189 (PMC8428527; doi:10.3389/fmicb.2021.712189)
Supplement: Supplementary Table 1 — Acid producing and morphological properties of selected lactic acid bacteria. [file Data_Sheet_1.docx]

**FIGURE S1| Production of sour porridge by natural fermentation.**

**FIGURE S2| pH of sour porridge.** pH change of sour porridge during 1-7 days of fermentation

**FIGURE S3| Scanning electron micrograph of natural and fermented** **starch granules. (A) natural** **starch granules (10 000×). (B) fermented starch granules (10 000×).**

| **TABLE S1 \| Acid producing and morphological properties of selected lactic acid bacteria.** | | | |
| --- | --- | --- | --- |
| **Strain** | **Individual form** | **pH** | **TA** |
| SZ01 | Bacillus | 3.89±0.02^a^ | 10.23±0.11^d^ |
| SZ02 | Bacillus | 3.94±0.05^a^ | 13.85±0.23^e^ |
| SZ03 | Bacillus | 3.88±0.01^a^ | 10.28±0.09^d^ |
| SZ11 | Bacillus | 4.11±0.05^b^ | 9.91±0.07^c^ |
| SZ25 | Bacillus | 4.35±0.04^d^ | 9.54±0.13^b^ |
| SZ26 | Bacillus | 4.25±0.04^c^ | 9.54±0.16^b^ |
| SZ38 | Bacillus | 4.14±0.04^b^ | 8.24±0.13^a^ |
| Means within a column followed by different letters are significantly different (*P*<0.05). | | | |
